# Assessment of Oxidative Stress Markers in Cats Undergoing Ovariohysterectomy by a Midline or Flank Approach

**DOI:** 10.1002/vms3.70363

**Published:** 2025-05-05

**Authors:** Zohreh Nazari, Mehrdad Mohri, Mohammad Heidarpour, Hossein Kazemi Mehrjerdi

**Affiliations:** ^1^ Department of Clinical Sciences Faculty of Veterinary Medicine Ferdowsi University of Mashhad Mashhad Iran

**Keywords:** antioxidants, ovariohysterectomy, oxidative stress, surgery

## Abstract

**Objectives:**

The main goal of this study was to assess oxidative stress markers in cats that were having an ovariohysterectomy (OVH) using either a midline (MAG) or flank (FAG) method.

**Methods:**

Twelve entire female cats were randomly assigned into two groups and ovariohysterectomized by a midline (MAG) or flank approach (FAG). Clinical evaluation was performed and haematological and biomarkers of oxidative status were evaluated at baseline (before surgery), one, and 24 h after endotracheal extubation.

**Results:**

Concerning cardiorespiratory parameters, there were no differences in heart rate (HR), respiratory rate (RR), rectal temperature (RT), peripheral capillary oxygen saturation (SpO2 saturation), and End‐tidal CO_2_ (ETCO_2_) values between groups or measured time during surgery. No significant differences were observed for the concentration of malondialdehyde (MDA), total antioxidant capacity (TAC), activities of erythrocyte glucose‐6‐phosphate dehydrogenase (G6PD), glutathione peroxidase (GPx) and superoxide dismutase (SOD) between the two groups. There were also no significant differences between different sampling times in each group.

**Conclusions and relevance:**

Results of the present study showed that ovariohysterectomy (OVH), either by midline or flank approaches, was not associated with oxidative stress and lipid peroxidation in the studied cats up to 24 h after surgery.

## Graphical abstract

This study evaluates oxidative stress markers in cats undergoing ovariohysterectomy (OVH) via midline (MAG) or flank (FAG) approaches. Results showed no significant differences in oxidative stress parameters malondialdehyde, total antioxidant capacity, glutathione peroxidase, superoxide dismutase, and glucose‐6‐phosphate dehydrogenase (MDA, TAC, GPx, SOD and G6PD) between groups. Both surgical techniques were physiologically comparable, indicating minimal oxidative stress up to 24 h post‐surgery

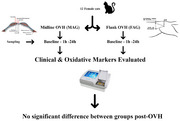

## 1 Introduction

Feline spaying or ovariohysterectomy (OVH) is one of the surgical procedures for sterilising female cats around the world. (Roberts et al. 2015, Munif et al. 2022) It can be done either through a midline or flank approach. (Murugesan et al. 2020) The lateral flank (FAG) approach is more common in the United Kingdom, and the midline (MAG) approach is more preferred in the United States of America. (Coe et al., 2006, May 2012) Benefits described for midline OVH include better visualisation and reduced postoperative pain scores. In contrast, a flank approach may offer shorter surgery time and reduced risk of hypothermia, herniation, or evisceration. (Coe et al. 2006) Evidence to support one approach over the other is limited. Comparative studies of various approaches to feline OVH, including midline and flank, have been performed. (Roberts et al. 2015, Coe et al. 2006, Burrow et al. 2006, Swaffield et al. 2020) Still, none of these studies attempted to assess the severity of the assessment of oxidative stress markers.

Reactive oxygen species (ROS) are metabolites produced during oxidative cellular metabolism. Low concentrations of ROS are necessary for physiological processes such as cell proliferation and differentiation, apoptosis, and cell‐mediated immunity. (Agarwal et al. 2005) Oxidative stress is an imbalance between ROS production and removal due to uncontrolled production of ROS, decreased antioxidant defences, or a combination of both. (Agarwal et al. 2005, Salzman et al. 2009) The critical role of oxidative stress in the pathogenesis of many diseases in humans and animals has been demonstrated (Szczubial et al. 2008), as well as many studies that established the involvement of ROS in surgical stress and complications. (Agarwal et al. 2005, Tsuchiya et al. 2008, Kang et al. 1998)

An increase in ROS can lead to oxidative damage (pathologic effects) to biomolecules, including nucleic acids, proteins, lipids, carbohydrates, or any other essential molecules. (Faramarzi et al. 2012) Protective mechanisms that neutralise the ROS and maintain free radicals in the physiologic range include an array of systemic enzyme and non‐enzyme antioxidant defences. (Agarwal et al. 2005) There are antioxidative enzymes glutathione peroxidase (GPx), superoxide dismutase (SOD), and glucose‐6‐phosphate dehydrogenase (G6PD). They must be balanced to prevent erythrocyte oxidative stress. (Edwards and Fuller 1996, Serin et al. 2008)

Oxidative stress is evaluated using biomarkers produced due to oxidative stress and measuring the activity of antioxidative enzymes. Lipids are the most susceptible macromolecule to oxidative damage due to the presence of unsaturated bonds. Malondialdehyde (MDA), the end product of lipid peroxidation, is an aldehyde produced following a ROS attack on the cell membrane's phospholipids. (Costa et al. 2024) The amounts of MDA demonstrate lipid peroxidation and cell exposure to oxidative stress. (Salzman et al. 2009, Serin et al. 2008, Marbut et al. 2009)

Proteins are key targets for oxidation reactions because they react quickly with oxidants and are abundant in cells, extracellular tissues, and bodily fluids. Furthermore, oxidative stress can lead to the degradation of lipids and carbohydrates into highly reactive intermediates, which can then attack proteins at various functional sites. This leads to forming a wide array of distinct posttranslational modifications through protein oxidation, glycoxidation, and lipoxidation (Kehm et al. 2021).

As an integrated parameter, serum total antioxidant capacity (TAC) can reflect all the antioxidants in plasma and body fluids. Therefore, the measurement of TAC will give an overview of the balance between oxidants and antioxidants in the plasma. (Szczubial et al. 2008, Camkerten et al. 2009) Studies showed that GPx, which catalyses the conversion of H_2_O_2_ to H_2_O, is crucial in preventing cell membranes from lipid peroxidation. (Pei et al. 2023)

It seems a reason for increasing ROS is the trauma of a surgical procedure. So, the less invasive procedure can lead to lower oxidative stress. (Tsuchiya et al. 2008, Stevens et al. 2019) However, if the stress response is prolonged, it results in more extended hospitalisation. Therefore, alleviating prolonged stress in surgical patients is essential for animal health. (Szczubial et al. 2008)

Numerous studies reported an increase in oxidative stress in both women and female laboratory animals after ovariectomy. Despite the large number of cats undergoing OVH annually, to the author's knowledge, there needs to be more data about evaluating antioxidative/oxidative status in OVH surgery in cats before, during, and following two different technical approaches. Therefore, this study aimed to compare the changes in oxidative stress and total antioxidant power status following flank or midline OVH in female cats.

## 2 Materials and Methods

### 2.1 Animals

All animal handling and methods received approval from the ethics council of Ferdowsi University of Mashhad. After obtaining informed consent, twelve privately owned female domestic short‐hair (DSH) breed cats aged between 6 months and 2 years were included in this study. Physical examination and haematological tests (blood counts, alanine aminotransferase, creatinine and total protein) were used to evaluate health status. Feline immunodeficiency virus (FIV) and feline leukaemia virus (FeLV) tests were performed on all included patients. Additionally, all animals had a history of routine deworming and vaccination according to standard veterinary guidelines. The exclusion criteria included systemic disease, pregnancy, lactation, and body weight < 2 kg. Cats were housed in individual cages within the cat ward of the Veterinary Teaching Hospital one day before the OVH. Patients were randomly divided into two groups based on the surgical technique: the flank access group (FAG; n = 6) and the midline access group (MAG; n = 6).

### 2.2 Anaesthesia

Cats fasted for 12 h before surgery with ad libitum access to water. The animals were premedicated with intramuscular injection of 0.1 mg/kg acepromazine maleate (Neurotranq 1%, Alfasan Co). After 20 minutes, a 24‐gauge catheter was inserted into a cephalic vein to provide medication and fluid therapy using Lactate Ringer's solution (5 mL/kg/hour) during surgery. Anaesthesia was induced with a combination of 0.25 mg/kg diazepam (Zepadic 10 mL/2 mL) and 6 mg/kg ketamine (Ketaset 10%, Alfasan Co) intravenously. After applying 0.1 mL of lidocaine hydrochloride (Serlix 10%, Kharaz Pharmaceutical Co, Iran) to the vocal cords, the cats were intubated with a lubricated endotracheal tube. Anaesthesia was maintained by isoflurane (Terrel, USA) 2–3% in 100% oxygen using a non‐rebreathing system. Vaporiser settings were adjusted to each cat's specific requirements, relying on observing heart rate (HR), respiratory rate (RR), palpebral reflex, globe position, pupil diameter, and reaction to surgical stimuli.

Patients received prophylactic antibiotics with cefazolin (AFAZOLE, 22 mg/kg, IV) and meloxicam (Rooyan Co., Iran, 0.2 mg/kg subcutaneous) before the induction of anaesthesia. A second administration of meloxicam was given 24 h later before hospital discharge. The operating room temperature was maintained between 25 and 27°C during the surgery. The same veterinary surgical team performed all the procedures in both groups.

### 2.3 Surgical Procedures

### 2.4 Midline Approach

Both midline and lateral approaches were performed according to the technique described by Coe et al. (2006). The cat was placed in dorsal recumbency, and a 2.5 cm incision was created in the skin, linea alba, and peritoneum in the midline, midway between the umbilicus and the pubis, caudally. The left uterine horn was exteriorised with a spay hook, and a hole was made in the broad ligament close to the ovary. The left ovarian pedicle was ligated with 3‐0 polyglactin 910 (Vicryl; Ethicon), transected using a double‐camp technique, and inspected to verify haemostasis before release. The procedure was repeated for the right ovary. The uterine body was exteriorised, and an encircling ligature of 3‐0 polyglactin 910 was placed cranial to the cervix and severed by using a triple‐clamp technique. The cervical stump was inspected carefully for haemorrhage and repositioned in its normal anatomic position. The celiotomy was closed in three layers. Linea alba and subcutaneous tissues were closed using a simple continuous pattern using 3‐0 polyglactin 910. A further intradermal continuous suture of 3‐0 polyglactin 910 was used to oppose the skin edges.

### 2.5 Flank Approach

The cat was placed in right lateral recumbence, and a 2.5 cm incision was created in the abdominal wall in a dorsoventral orientation starting from the third angle of a triangle defined by the greater trochanter of the femur and the wing of the ilium. Upon accessing the peritoneum, the subsequent steps were the same as those outlined for the midline approach. The internal and external muscle layers were closed together, and subcutaneous tissue was closed using a simple continuous pattern with 3‐0 polyglactin 910. The skin was sutured with an intradermic pattern.

### 2.6 Postoperative Care

The Duration of surgery (from the first incision until the placement of the last suture), anaesthesia (from the injection of acepromazine to turning off the vaporiser dial), and time to extubation (from turning off the vaporiser dial until extubation) were recorded for each cat. Extubation took place once the palpebral reflexes had returned. Heart rate (HR; beats/minute), respiratory rate (RR; breaths/minute), rectal temperature (RT; °C), peripheral capillary oxygen saturation (SpO_2_ saturation), and end‐tidal CO2 (EtCO_2_) were assessed in each cat with a multiparametric monitor (cardio set, ARAD P10, SaIran Medical Industrial, Iran) at five min intervals during anaesthesia. In addition, HR, RR, and RT were also measured preoperatively as baseline data.

### 2.7 Blood Samples Collection

Blood samples (4 mL) were collected via the jugular vein before surgery (baseline) and one and 24 h after endotracheal extubation. Each sample was transferred into two tubes, one containing Ethylenediaminetetraace (EDTA) as an anticoagulant for preparing red blood cells (RBCs) haemolysis and the other without an anticoagulant for separating serum. Blood was permitted to coagulate at ambient temperature for 30 min, and then the serums were separated by centrifuging the blood at 3500 g for ten min. Then, all the serum was aliquoted into 1.5 mL plastic microtubes and stored at ‐20°C until MDA and TAC analyses. For preparing RBC haemolysis, tubes containing EDTA anticoagulant were centrifuged at 2500 g for five min. Supernatant plasma was collected, and an equal volume of 0.9% sodium chloride solution was added. This step was repeated three times. The last time, 0.9% sodium chloride solution was discarded, and an equal volume of cold distilled water was added and placed in the refrigerator for 15 min. Then, samples were aliquoted into 1.5 mL plastic microtubes and stored at ‐20°C until the levels of SOD, GPx and G6PD were analysed.

### 2.8 Measurement of MDA

Serum MDA was measured in this study using the spectrophotometric method of Placer and colleagues. (Placer et al. 1966) The reaction mixture consisted of 0.2 mL of serum, 1.3 mL of 0.2 M Tris–0.16 M KCl buffer (pH 7.4), and 1.5 mL of thiobarbituric acid reagent. The mixture was heated in a boiling water bath for ten min. After cooling, 3 mL of pyridine/nbutanol (3:1, v/v) and 1 mL of 1 N sodium hydroxide were added and mixed by vigorous shaking. A blank was run simultaneously by incorporating 0.2 mL of distilled water instead of the serum. The absorbance of the test sample was read at 548 nm. The nmol of MDA per ml of serum was calculated by using the following formula: MDA (nm/ml) = (V^×^OD548)/0.152

### 2.9 Measurement of Antioxidant Enzyme Activity

The activity of GPx in erythrocytes was estimated using a Ransel test kit (Randox Laboratories Ltd. G.B.) based on the method described by Paglia and Valentine. (Paglia and Valentine 1967) The activity of SOD was estimated using the modified Iodophenyl Nitrophenol Phenyl Tetrazolium Chloride method by use of a Ransod test kit (Randox Laboratories Ltd. G.B.), and the activity of G6PD was estimated using a G6PD test kit (Randox Laboratories Ltd., G.B.). TAC of the blood serum was determined based on the suppressing activity of antioxidants in producing a coloured radical cation. A commercial test kit (TAS test kit, Randox Laboratories Ltd., G.B.) was used for serum TAC measurement.

### 2.10 Hematological Analysis

Haematological parameters in blood samples containing anticoagulants were performed using an automatic haematology analyser (Celltac Automated Haematology Analyser MEK 6450, Nihon Kohden, Japan). Haemoglobin (HGB), haematocrit (HTC), total RBC, total white blood cell (WBC), mean corpuscular volume (MCV), mean corpuscular haemoglobin (MCH), mean corpuscular haemoglobin concentration (MCHC), and platelet count were evaluated.

### 2.11 Statistical Analysis

The results were analysed by using SPSS software (version 26). The Shapiro‐Wilk test was used to test the data for a normal distribution. Because data were normally distributed, parametric analysis was used. Demographic data and procedural times were assessed using the independent‐samples t test. The comparison among data related to the midline and flank approach was performed using repeated measures ANOVA and Dunnett's supplementary test with *p* < 0.05 set as the significance limit. All values are reported as means ± SE. The effects of time, group, and interaction between time and group were evaluated.

## 3 Results

We observed no complications following OVH surgery in cats, and recovery from anaesthesia was smooth. The sample characteristics by group are described in Table 1. The groups were considered to be homogeneous in terms of weight and age. The mean body weight of cats was 3.13 ± 0.044 kg, and the mean age was 15.16 ± 1.64 months (Table 1). No significant differences between the two groups were observed in the duration of surgery, anaesthesia, and extubation time. The data presented in Table 2 reveal that all the physiological indices were almost stabilised. Time significantly affected the HR, RR and RT (*p* < 0.05); however, the group did not significantly affect the parameters. SpO_2_ was 98/100%, showing good tissue oxygenation.

**TABLE 1:** Weight, age and procedural times (mean ± standard errors) of cats undergoing ovariohysterectomy with (MAG) or (MAG) approach.

	Midline	Flank	P Value
Weight (months)	3.05 ± 0.63	3.20 ± 0.05	0.34
Age (kg)	15.61 ± 2.43	16.75 ± 2.34	0.89
Surgery time (min)	21.46 ± 0.84	20.78 ± 0.78	0.98
Anaesthesia time (min)	46.46 ± 0.84	47.01 ± 0.49	0.56
Extubation time (min)	7.48 ± 0.32	7.40 ± 0.43	0.46

**TABLE 2:** Changes in physiological parameters of cats undergoing ovariohysterectomy with (MAG) or (FAG) approach.

Physiological parameters	Groups		*p* value		
	Midline	Flank	Time	Group	Time group*
HR (beats/min)	2.58± 134.60	2.59±135.91	0.001	0.727	0.836
RR (breaths/min)	0.717±26.687	0771±26.563	0.001	0.904	1.00
RT (°C)	0.065±37.55	0.065± 37.51	0.001	0.947	0.831
SpO_2_	106±98.33	108± 98.29	0.058	0.879	0.835
EtCO_2_	0.774± 40.44	0.874±40.25	0.974	0.865	0.983

**Abbreviations**: EtCO_2_, end‐tidal CO_2_.; HR, heart rate; RR, respiratory rate; RT, rectal temperature; SpO_2,_ peripheral capillary oxygen saturation.

### 3.1 Oxidative Stress Parameters

**FIGURE 1:**
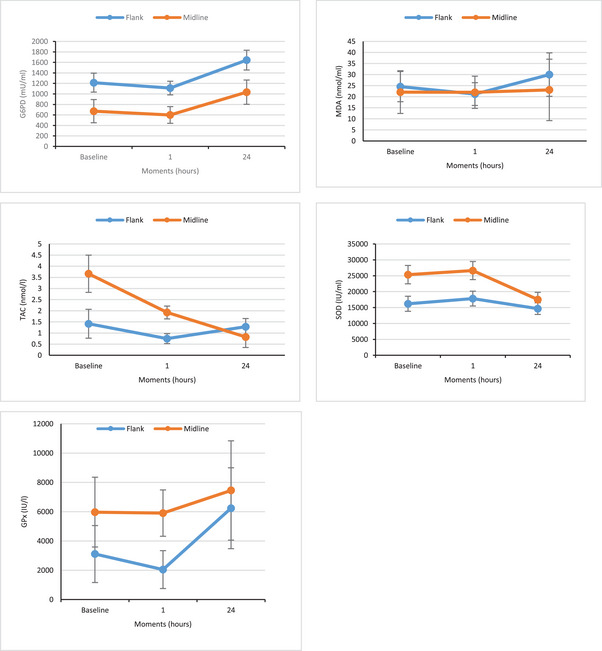
Mean ± SE of MDA, GPx, SOD, TAC and G6PD concentration at baseline, 1 and 24 h after operation in cats undergoing ovariohysterectomy by flank or midline approach. **Abbreviations**: G6PD, glucose 6 Phosphate Dehydrogenase; GP_X_, glutathion peroxidase; MDA, malondialdehyde; SOD, superoxide dismutase; TAC, total antioxidant capacity.

The mean values of MDA, TAC, SOD, G6PD and GPx in two groups and at different times are presented in Table 3 and Figure 1. Time showed a significant effect on the amounts of TAC, G6PD and SOD (*p* < 0.05); however, the group did not show any significant effect on the parameters. Although mean serum TAC and SOD were higher and erythrocyte G6PD was lower in cats in the MAG compared to those in the FAG, these differences were not significant.

**TABLE 3:** The amounts of oxidative stress parameters in different groups (mean ± standard error).

Oxidative stress parameters	Groups		*p* value		
	Midline	Flank	Time	Group	Time group*
MDA (nmol/ml)	9.578± 22.40	6.772± 25.268	0.604	0.814	0.736
TAC (mmol/l)	0.337± 2.137	0.261±1.149	0.032	0.060	0.065
SOD (IU /ml)	2389.19±23180.00	1950.76± 16230.00	0.001	0.054	0.061
G6PD (mU/ml)	188.205± 768.41	153.66± 1324.00	0.000	0.052	0.873
GPx (IU/l)	2159.99± 6438.00	1763.63±3794.00	0.174	0.371	0.692

**Abbreviations**: G6PD, glucose 6 Phosphate Dehydrogenase; GP_X_, glutathion peroxidase.;MDA, malondialdehyde; SOD, superoxide dismutase; TAC, total antioxidant capacity.

### 3.2 Haematologic Parameters

The mean and SE of HGB, HTC or PCV, RBC, WBC, MCV, MCH, MCHC and platelets in different groups are presented in Table 4. Time showed a significant effect on the amounts of HGB, HTC, and total RBC (*p* < 0.05), and the group showed a significant effect on MCHC (*p* < 0.05). The interaction between time and group was insignificant for all parameters (*p* > 0.05).

**TABLE 4:** The effects of OVH surgery on hematologic parameters through two approaches (mean ± standard error).

Haematologic parameters	Groups		P Value		
	Midline	Flank	Time	Group	Time group*
PCV (%)	2.349 ±29.950	1.918± 32.250	0.005	0.470	0.918
Hb (g/dl)	0.988± 12.075	0.807 ±11.039	0.002	0.440	0.686
RBC (×10^6^/µl)	0.648 ±7.102	0.529± 6.782	0.003	0.712	0.840
MCV(fl)	3.803 ±41.942	3.105 ±48.767	0.687	0.202	0.909
MCH(pg)	1.025 ±16.925	0.837 ±16.644	0.561	0.837	0.658
MCHC(g/dl)	1.755 ±40.533	1.433 ±34.456	0.448	0.028	0.853
Total WBC(/µl)	4185.88± 18790.00	3417.75± 16620.00	0.699	0.699	0.561
Plt(×10^5^/µl)	0.602± 2.089	0.492 ±2.166	0.965	0.924	0.691

**Abbreviations**: Hb, haemoglobin; MCH, mean corpuscular haemoglobin; MCHC, mean corpuscular haemoglobin concentration; MCV, mean corpuscular volume;PCV, packet cell volume; Plt, platelet; RBC, total red blood cell; WBC, total white blood cell.

## 4 Discussion

This clinical study compared oxidative stress markers between midline and flank approaches for OVH in cats. The intensity of oxidative stress was analysed by measuring the amounts of MDA, TAC and antioxidant enzymes such as SOD, GPx and G6PD in erythrocytes. The results of this research indicate that the oxidative stress response in cats undergoing OVH shows no significant differences between the MAG and FAG.

The absence of differences in HR, RR, RT, SpO_2_ saturation and EtCO_2_ levels among the groups indicates that both methods are physiologically similar regarding perioperative stability. These parameters were also within normal limits during surgery. This is consistent with earlier research indicating no major differences in clinical parameters during midline OVH surgeries in cats. (Carbonari et al. 2025)

Regardless of technique, prolonged surgical times are associated with increased oxidative stress and inflammatory responses due to extended tissue manipulation and anaesthesia exposure. (Tsuchiya et al. 2008, Del Romero et al. 2020) There were no notable variations in the length of surgery and anaesthesia between the two groups, further reinforcing the similarity of both surgical methods regarding procedural efficiency and patient stability. Various studies have conflicting reports on the duration of midline and flank OVH. As Coe et al. reported, the skin incision's duration to enter the peritoneum is longer with the FAG, and uterus finding will take longer with the MAG. However, the total duration of surgery was similar in both groups. (Coe et al. 2006) Some studies have shown that surgical time was shorter in the flank approach than in the midline approach, which has been attributed to the surgeon's experience with the flank approach. (Carbonari et al. 2025)

During surgical procedures, oxidative stress may occur due to tissue trauma, inflammation, hypoxia‐reoxygenation events, and the release of inflammatory cytokines. Blood contains many antioxidant molecules that prevent the formation of free radicals and remove free radicals, which protect the body during exposure to them. The serum levels of each antioxidant can be measured separately, but this method is time‐consuming and expensive. On the other hand, the TAC of the blood indicates its antioxidant status and can be easily evaluated. (Camkerten et al. 2009, Collins 2004) In this study, the TAC of the serum in the MAG showed a tendency to increase compared to the FAG (*p* = 0.060). Still, comparing the two groups at different times separately showed no significant difference. Also, statistical differences were not found between the pre‐and post‐ operation animals. These results are in accordance with previous studies, which showed that TAC did not show any significant difference within all groups of dogs after OVH. (Gautier et al., 2020) Also, Szymczyk et al. showed no significant difference in serum ferric ion (as an indicator of total blood serum antioxidant status) after hysterectomy surgery without closing the peritoneum. (Szymczyk et al. 2003)

SOD is an enzyme containing copper and zinc inside red blood cells, which converts two oxygen‐free radical molecules into hydrogen peroxide and oxygen. Although this enzyme protects against oxidative damage, it increases oxidative damage when hydrogen peroxide catabolism is compromised. (Pei et al. 2023) In this study, no significant difference was seen between and within groups. Our results are similar to those previously described, where the activity of SOD and CAT did not decrease after midline OVH in cats. (Teixeira et al., 2020) Similar data were also reported on sterilisation surgery in dogs with two laparoscopic methods (vasectomy) and open castration under xylazine‐ketamine anaesthesia. (Mahalingam et al. 2009) Contrary to these studies, Godin and Garmett reported that the activity of the myocardial SOD enzyme was significantly higher in rats after anaesthesia with halothane compared to carbon dioxide. (Godin and Garnett 1994) This research indicates that the anaesthesia protocol may also influence the oxidative stress process besides the surgical technique.

GPx is a major intracellular nonenzymatic antioxidant involved in several defence processes against oxidative damage. GPx synthesis is regulated by oxidants, antioxidants, and inflammatory and anti‐inflammatory agents. (Pei et al. 2023, Irato and Santovito 2021) The absence of variations in GP_X_ levels in our research aligned with findings from other studies, which demonstrated that the GP_X_ concentration across all groups did not exhibit any significant differences until one day following OVH surgery in dogs. (Salavati et al. 2021) A study found that OVH in juvenile female dogs can induce oxidative stress and decrease tocopherol (vitamin E) levels, which protect cell membranes. (Costa et al. 2024) In our study, the lack of significant depletion of antioxidant markers (TAC, GPx, SOD) within 24 h after OVH suggests that the oxidative stress from this surgery might not be sufficient to affect antioxidant reserves. This difference may be attributed to species‐specific responses and variations in surgical trauma.

Any disturbances in production and reduction of ROS can lead to oxidative stress. Oxidative stress develops when oxygen and nitrogen free radicals exceed the capacity of the antioxidant defences of the organism and are associated with different diseases in humans and veterinary medicine. The result of the reactions of ROS with biomolecules is the formation of substances that can be used as markers of oxidative damage, such as MDA. (Edwards and Fuller 1996, Marbut et al. 2009) Determining the level of plasma MDA is the most sensitive and commonly used method for estimating the level of lipid peroxidation. (Serin et al. 2008) Studies showed an increase in the level of lipid peroxidation after abdominal surgeries such as human hysterectomy (Szymczyk et al. 2003, Sane et al. 1993) and general anaesthesia in dogs. (Serin et al. 2008, Naziroǧlu and Günay 1999)

In this study, no statistically significant difference was observed in the level of serum MDA between groups or measured time during the study (*p* > 0.05). Our data is consistent with those of Teixeira et al., who reported no increase in the MDA value after midline OVH in cats. (Teixeira et al. 2020). Also, Yilmaz et al. showed no significant difference after OVH surgery in dogs anaesthetised by xylazine and ketamine. (Yilmaz et al., 2014)

In contrast, some studies have shown that dogs' MDA levels increase following OVH. (Costa et al. 2024). Serin et al. and Gunay et al. observed a significant increase in MDA levels 24 h after OVH surgery in dogs that were anaesthetised by a xylazine and ketamine combination in approximately 60 min. (Serin et al. 2008, Gunay et al. 2011) Sakundech et al. (2020) found that MDA levels reach their highest point on the third day following surgery due to inflammation caused by surgical trauma, and they recommend that dogs undergoing this procedure should be given antioxidants. They attributed this phenomenon to a decrease in antioxidant capacity on the third day post‐OVH. (Sakundech et al., 2020) Also, increased serum MDA was observed in women in the initial stages of hysterectomy surgery. (Sane et al. 1993) As mentioned before, similar to Yilmaz et al., serum MDA did not show any significant difference within the two groups in our study. This difference between results might be related to the anaesthetic protocol and duration of surgery, which was shorter than in previous studies. (Yilmaz et al., 2014)

Furthermore, a study examining the effects of OVH on oxidative stress markers in female dogs found that surgical intervention led to transient increases in oxidative stress indicators, including MDA, and reductions in antioxidant enzyme activities. However, these effects were temporary and returned to baseline within a short period post‐surgery. (Szczubial et al. 2015) These findings support the idea that surgical trauma can induce oxidative stress, but the body's antioxidant defence mechanisms can effectively compensate, preventing long‐term oxidative damage. This aligns with our results, where oxidative stress markers did not show significant variation between groups or time points, suggesting that the oxidative stress induced by OVH in cats may be mild and rapidly regulated by endogenous antioxidant systems. (Szczubial et al. 2008)

The increase of oxidative stress in open abdominal surgery has not been proven well. Opening the abdominal wall and briefly manipulating the intestines have caused oxidative damage in laboratory animals. In laparotomy, abdominal viscera are constantly exposed to many oxygen molecules, which will lead to oxidative stress. Oxidative stress during surgery is clinically important, so reducing this stress as much as possible will be helpful in the recovery process after surgery. (Tsuchiya et al., 2008) The present study was the first to compare oxidative stress in cats following OVH with flank and midline approaches, and the results show no difference between these two methods.

### 4.1 There are Several Limitations in This Study

A major limitation was that oxidative stress assessments were performed shortly after surgery. One study showed that on day 14 after OVH in bitches, the total antioxidant power was higher than during preoperative, three h after starting OVH, and on days three, seven and ten. (Lee and MC 2014) Therefore, the amount of oxidative stress should be checked for longer after the surgery. Also, increasing the sample size in each group will help achieve better and more reliable results. Although this is a major limitation, some veterinary studies have also used fewer animals (6, (Lee and MC 2014), 8 (Hancock et al. 2005), 10 (Freeman et al. 2010), 55 (Del Romero et al. 2020), 3 (Davidson et al. 2004)).

## 5 Conclusion

In conclusion, our research indicates no notable differences in oxidative stress indicators when comparing the midline and flank methods within 24 h following OVH in cats. These results offer important information about the physiological effects of various surgical techniques and imply that both methods are equally safe regarding oxidative stress and the stability of the perioperative period.

## Author Contributions

Conceptualisation: Zohreh Nazari, Hossein Kazemi Mehrjerdi, Mehrdad Mohri and Mohammad Heidarpour. Methodology: All Authors. Software: Hossein Kazemi Mehrjerdi, Mehrdad Mohri and Mohammad Heidarpour. Validation: Zohreh Nazari, Hossein Kazemi Mehrjerdi and Mohammad Heidarpour. Formal analysis: Mohammad Heidarpour. Investigation: All Authors.

## Conflicts of Interest

The authors declare no conflict of interest.

## Ethics Statement

The study protocol was assessed by the Research Committee of the Faculty of Veterinary Medicine and approved by the Research Ethics Committee of Ferdowsi University of Mashhad, Mashhad, Iran (Approval ID: IR.UM.REC.1400.331). Informed consent (verbal or written) was obtained from the owner of all animals described in this work for all procedures undertaken (prospective studies).

### Peer Review

The peer review history for this article is available at https://www.webofscience.com/api/gateway/wos/peer‐review/10.1002/vms3.70363.

## Data Availability

The data that support the findings of this study are available from the corresponding author upon reasonable request.
